# Optical Sensors for Multi-Axis Angle and Displacement Measurement Using Grating Reflectors

**DOI:** 10.3390/s19235289

**Published:** 2019-12-01

**Authors:** Yuki Shimizu, Hiraku Matsukuma, Wei Gao

**Affiliations:** Precision Nanometrology Laboratory, Department of Finemechanics, Tohoku University, Sendai 980-8579, Japan; hiraku.matsukuma@nano.mech.tohoku.ac.jp (H.M.); gaowei@cc.mech.tohoku.ac.jp (W.G.)

**Keywords:** optical sensor, multi-axis angle and displacement measurement, dimensional metrology, planar encoder, planar scale grating, interference lithography

## Abstract

In dimensional metrology it is necessary to carry out multi-axis angle and displacement measurement for high-precision positioning. Although the state-of-the-art linear displacement sensors have sub-nanometric measurement resolution, it is not easy to suppress the increase of measurement uncertainty when being applied for multi-axis angle and displacement measurement due to the Abbe errors and the influences of sensor misalignment. In this review article, the state-of-the-art multi-axis optical sensors, such as the three-axis autocollimator, the three-axis planar encoder, and the six-degree-of-freedom planar encoder based on a planar scale grating are introduced. With the employment of grating reflectors, measurement of multi-axis translational and angular displacement can be carried out while employing a single laser beam. Fabrication methods of a large-area planar scale grating based on a single-point diamond cutting with the fast tool servo technique and the interference lithography are also presented, followed by the description of the evaluation method of the large-area planar scale grating based on the Fizeau interferometer.

## 1. Introduction

Measurement of angle and displacement is a fundamental activity in various areas of dimensional metrology [1,2,3]. In a precision positioning system, it is necessary to carry out multi-axis angle and displacement measurement for accurately specifying the position of a target in a plane or in three-dimensional (3D) space [4]. For this purpose, multi-axis coordinate measurement methods based on the multi-axis Cartesian coordinate system, the multi-axis polar coordinate system, the triangulation system or the multilateration system are employed [4,5]. The target position with respect to the origin of a given coordinate system can be determined by employing several linear displacement sensors and/or angle sensors. It should be noted that the angles obtained by angle sensors such as optical rotary encoders will be employed in the measurement methods based on the multi-axis polar coordinate system and the triangulation system. Meanwhile, the increase of the uncertainty of position measurement cannot be avoided when the measured angles are employed for specifying the target position in a plane or in 3D space, since the error in angle measurement could be amplified with the increase of the distance between the target and each of the angle sensors. Therefore, measurement methods based on the multi-axis Cartesian coordinate system, which are the main focus of this review article, are often employed for the applications requiring high positioning accuracy [4,6]. The most straightforward method for specifying the position of a target in a plane or in 3D space based on the multi-axis Cartesian coordinate system is to employ orthogonally placed multiple linear displacement sensors such as laser interferometers [6,7] and/or linear encoders [8,9]. Capacitive displacement sensors [10], high-precision strain gauges [11,12] and fiber sensors [13] can also be employed when the required measurement range is up to several-tens of micrometers.

One of the main features of the laser interferometers, in which the wavelength of light is employed as the ruler for displacement measurement, is the easiness of use for designing a multi-axis measurement system [14,15]. By designing the optical setup in such a way that each of the orthogonally placed axes of laser interferometers coincides with the corresponding motion axis of the target, the Abbe errors [16,17], which is one of the main contributors in measurement uncertainty for precision positioning [4], can be minimized [18,19,20]. It is also easy to realize displacement measurement traceable to the primary standard of length [20,21]. However, on the other hand, displacement measurement based on laser interferometers can be affected by the change of the refractive index of air caused by environmental disturbances such as the changes in temperature, humidity, and air pressure [21]. Although many efforts have been made so far [4,22,23,24,25,26,27], the influence of the refractive index error is a major concern when employing laser interferometers in a multi-axis measurement system. In addition, the alignment of the multiple laser beams is a time-consuming task. On the contrary, linear encoders are relatively strong against environmental disturbances. Among several types of linear encoders [28,29,30,31], optical linear encoders can achieve a high resolution and good stability due to the short grating pitch and a short optical path length [4,28]. Although it is difficult to assure high accuracy because there are some critical issues such as a periodic error, a distortion error, and a thermal error, optical linear encoders are often employed in precision positioning systems in machine tools and measuring instruments due to their high resolutions and easy-to-use designs. Many efforts have been made so far to improve the resolution of optical linear encoders [28,32,33,34,35,36,37,38,39,40,41,42], and nowadays state-of-the-art optical linear encoders can realize a sub-nanometer resolution comparable to that of the laser interferometers [4,6,7]. Furthermore, absolute linear encoders enable machine tools, measuring instruments and fabrication equipment to be operated without the initialization of their positioning systems [43], which is mandatory when the incremental laser interferometers and/or linear encoders are employed. Due to the improvements in the resolution and bandwidth of such absolute linear encoders, in recent years, the share of optical linear encoders is increasing [4,44]. Meanwhile, due to the principle of the linear encoder where the scale graduations are read by the reading head placed with respect to the scale with a short working distance, it is difficult to align the measurement axis of the linear encoder to coincide with the corresponding motion axis of the object. As a result, it is difficult to avoid the Abbe error that will be generated in accordance with the angular motion errors of the target of interest [2,19].

One of the solutions for the above issues is to measure the angular motion errors and compensate for their influences by using high-precision angle sensors in the measurement system [2,45]. For this purpose, high-precision compact angle sensors capable of detecting dynamic angular motion/displacement are required. In responding to the above requirements, two-axis optical angle sensors [46,47,48,49,50,51] based on the laser autocollimation [51], as well as the three-axis autocollimator [52], have been developed. The applications of planar encoders or surface encoders [53,54,55,56,57,58,59,60,61,62,63,64,65,66,67,68,69,70,71,72,73,74,75,76,77,78,79], which can carry out simultaneous detection of the position in a plane or in 3D space with the enhancement of a planar scale grating, is another possible solution. The planar encoders/surface encoders are expected to achieve multi-axis angle and/or displacement measurement, while complying with the Abbe principle and avoiding the increase of measurement uncertainty due to the increase of the degrees of freedom.

In this review article, optical sensors for measurement of multi-axis angle and displacement are treated. In Section 2, optical angle sensors for measurement of multi-axis small angular displacements/motions of a target of interest in a positioning system are introduced, while focusing on the optical angle sensors based on the laser autocollimation [47,51]. In addition, the principle of the three-axis autocollimator [52] that can carry out three-axis angle measurement by using a single measurement laser beam with the enhancement of a reflective diffraction grating is introduced. In Section 3, optical displacement sensors for measurement of multi-axis displacement of a target of interest in a positioning system are described. After the brief introduction of conventional multi-axis measurement systems based on laser interferometers and/or linear encoders, principles of surface encoders / planar encoders capable of detecting the multi-axis displacement in a plane or in 3D space [53,56,57,58,59] with the employment of a planar scale grating are introduced. In addition, the six-degree-of-freedom planar encoder [54,55], which can be realized by combining the principle of three-axis autocollimator and that of the three-axis planar encoder, is introduced. Furthermore, in the following section, the fabrication method of large-area planar scale gratings based on the fast-tool-servo technique [80,81], the non-orthogonal two-axis Lloyd’s mirror interferometer [82,83,84,85], as well as the evaluation technique of the large-area planar scale gratings based on the Fizeau interferometer [86,87,88] are presented.

## 2. Multi-Axis Optical Angle Sensors

### 2.1. Conventional Optical Sensors for Multi-Axis Angle Measurement

As described in the Introduction, high-precision optical angle sensors capable of detecting dynamic angular motion of a target of interest must compensate for the influences of Abbe errors in multi-axis coordinate measurement methods based on the multi-axis Cartesian coordinate system [2,4]. In the case of the measurement system employing laser interferometers, the dynamic angular motion can be detected by employing additional laser interferometers in the measurement system [89]. For example, by placing an additional laser interferometer (output: *X*_2_) for measurement of the *X*-displacement with the *Y*-directional offset Δ*d_Y_* with respect to the original *X*-directional laser interferometer (output: *X*_1_), the angular displacement about the *Z*-axis (Δ*θ*_Z_) can be obtained as Δ*θ_Z_*=arctan((Δ*X*_2_-Δ*X*_1_)/Δ*d_Y_*). In the same manner, the angular displacements about the *X*- and *Y*-axes can be measured by adding measurement laser beams. However, it is a time-consuming task to carry out precision alignments of the multiple laser beams and the reflective mirrors in a positioning system. Furthermore, a well-controlled environment for suppressing the refractive index error is necessary for accurate measurement [22,23,24,25,26,27]. On the contrary, optical angle sensors, in which the angular displacement of a target of interest is measured by detecting the angle of reflectance of the reflected light, can carry out high precision angular displacement measurement by using a simple optical setup.

**Figure 1:**
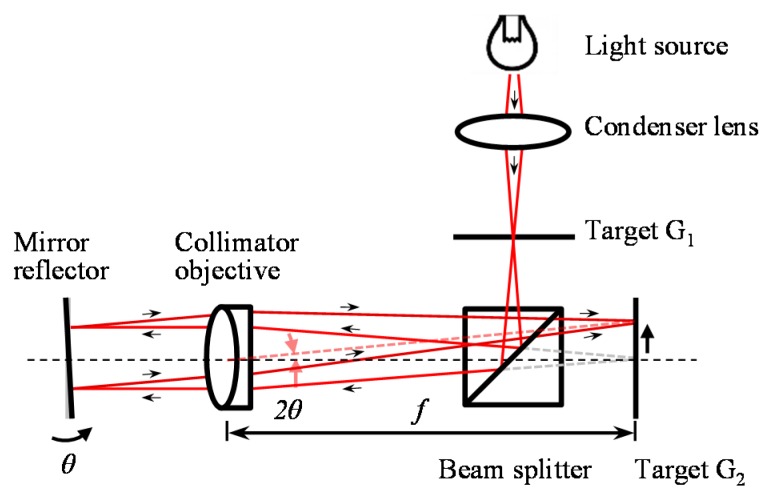
Optical angle sensor based on the autocollimation method.

Several types of angle sensors based on the optical lever method [2,90], the critical angle method [91], the optical fiber-based method [92] or the autocollimation method [2,93] have been developed so far. Among them, the angle sensors based on the autocollimation method are suitable for the evaluation of the angular displacements of a multi-degree-of-freedom stage system, since the distance between a measurement target and the reading head will not affect the measurement of the angular displacements. Simple and compact optical setups are another advantage of the angle sensors based on the autocollimation method. Figure 1 shows the optical setup of an optical angle sensor based on the autocollimation method [94]. As can be seen in the figure, a pair of targets (G_1_ and G_2_) is placed at the conjugating front and focal planes of an objective lens, respectively. G_1_ is imaged on the plane of G_2_ through the objective lens having the focal length *f*. With a small angular displacement Δ*θ* of the reflective mirror, the position of the image of G_1_ shifts 2*f*Δ*θ* with respect to the optical axis of the objective lens; namely, the angular displacement Δ*θ* of the mirror reflector can be converted into the image displacement on a back focal plane of the objective lens. In most of the cases, in recent years, photoelectric autocollimators having photodetectors are employed. In the case of the autocollimator having a two-dimensional (2D) CCD (charge-coupled device) sensor or a position-sensitive detector (PSD) as the photodetector, the two-dimensional angular displacement of a mirror reflector can be detected simultaneously. In addition, in recent years, a LED (light-emitting diode) is often employed as the light source for the photoelectric autocollimator, instead of conventional light sources such as a halogen lamp. Most of the commercially-available autocollimators have a resolution of up to 0.01 arc-second with a measurement range from several-ten arc-seconds to over 2000 arc-seconds. In addition, a high resolution of better than 0.005 arc-second has already been achieved by some commercial products [48]. It should be noted that, in the case of the optical angle sensors employing a 2D CCD or a 2D PSD, what the angle sensor can detect is the angular displacement of a target of interest or low-frequency motion due to the low-frequency bandwidth of the photodetector. It is therefore difficult to carry out the detection of the dynamic angular motion of the target with high-frequency components, which is required to compensate for the influence of Abbe errors in the multi-axis displacement measurement. In addition, the size of an optical angle sensor based on the autocollimation method becomes large when it is designed to have a high resolution, which is proportional to the focal length of a collimator objective employed in the optical setup [2].

**Figure 2:**
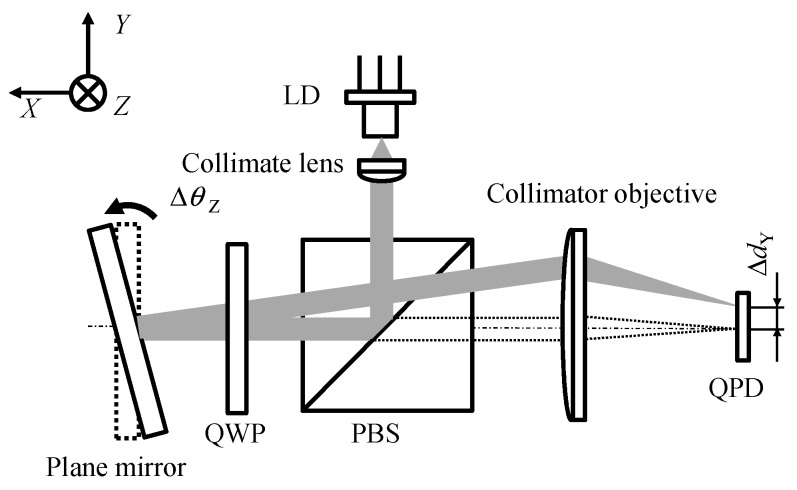
Optical angle sensor based on the laser autocollimation.

Meanwhile, the optical angle sensors based on the laser autocollimation, in which a laser and photodiodes are employed as the light source and the photodetectors, respectively, can realize the high-precision measurement of the dynamic angular motion [4,51]. Figure 2 shows the setup of an optical angle sensor based on the laser autocollimation [51], in which a laser diode (LD) and photodiodes are employed as the light source and the detectors, respectively, suitable for measurement of small angular displacements and dynamic angular motion in a positioning system [2]. For the sake of simplicity, the one-dimensional angular displacement/motion of a mirror reflector about the *X*-axis is treated in the figure. The laser beam emitted from LD is collimated by a collimating lens, and is projected onto a mirror reflector through a polarizing beam splitter (PBS) and a quarter-wave plate (QWP). The laser beam reflected by the mirror reflector passes through QWP again, reflected by PBS, and is focused onto a photodetector by the collimator objective with the focal length *f*. It should be noted that PBS and QWP act as an optical isolator. 

When the normal of the mirror reflector has an angular displacement Δ*θ* with respect to the projected laser beam, the focused laser beam on the photodetector will have a displacement *δ* that can be described by the following equation [2]:

(1) δ=ftan(2Δθ)

Since *f* is a known design parameter, Δ*θ* can be obtained by detecting *δ* with the photodetector. It should be emphasized that the detection of Δ*θ* will not be affected by the working distance of the optical setup, as can be seen in Equation (2); this feature is different from that of the optical angle sensors based on the optical lever technique [2,90], and is suitable for the evaluation of an angular motion error of a target having a long travel range such as a long-stroke linear slide.

In addition, the employment of a four-cell, two-cell or single-cell photodiode instead of two-dimensional sensors such as a 2D CCD or a 2D PSD makes the sensitivity of optical angle sensor independent from the focal length *f* of the collimator objective [2]. Now we assume that the focused laser beam on the photodetector has uniform light intensity distribution, for the sake of simplicity. Denoting the diameter of the collimated laser beam made incident to the collimator objective as *D*, and that of the focused laser beam as *d*, the output *H*_Out_ from the angle sensor can be described as follows [2]:

(2) $$H_{\mathrm{Out}}=\frac{8\delta }{\pi d}\times 100\;[\%]$$

Assuming that the collimator objective is an ideal lens without aberrations, the following relationship can be found between *D* and *d*:

(3) d=2.44fλ/D

From Equations (1), (2) and (3), the following equation can be found:

(4) $$H_{\mathrm{Out}}\propto \left(D/\lambda \right)\Delta \theta$$

The above equation indicates that the sensor sensitivity is independent of the focal length *f* of the collimator objective; this fact means that the setup of the optical angle sensor based on the laser autocollimation can be designed in a compact size by employing a collimator objective having a short focal length [2,46,95]. It should be noted that the measurement range of the optical angle sensor depends on the diameter *d* of the focused laser beam on a photodiode. Meanwhile, by employing a photodiode having multiple cells, the measurement range can be expanded without sacrificing the resolution [49]. Furthermore, by employing a measurement laser beam having a large diameter *D* and single-cell photodiodes, a high resolution better than 0.001 arc-second can be achieved [47]. The two-axis laser autocollimator has also been applied for surface topography measurement [96,97]. It should be noted that the applications of above mentioned optical angle sensors having a laser source are limited for measurement of mirror-finished surfaces or the angle of a target capable of mounting a mirror reflector due to coherence matters such as speckle and diffraction.

### 2.2. Three-Axis Autocollimator Using Diffraction Grating Reflector

The above described optical angle sensors, in which a flat mirror is employed as the target reflector for angle measurement, can carry out two-axis angle measurement. However, they cannot detect the angular displacement of the mirror reflector about the laser beam axis, in principle. This can be measured by employing a pair of orthogonally arranged two-axis optical angle sensors. However, the measurement setup becomes large and costs too much. Several methods have therefore been developed so far to carry out three-axis angle measurement by using a single laser beam [98,99]. For example, a method employing a target reflector having a leaf spring mechanism has realized the measurement of the three-axis angular displacement while employing a conventional photoelectric two-axis autocollimator [98], although the system has suffered from the mechanical instability of the target reflector. A method has also been developed based on the optical lever method, while employing a reflective-type diffraction grating as the target reflector for angle measurement [99]. In the method, angular displacement about the laser beam axis is detected by monitoring the diffracted beams. Meanwhile, since the method is based on the optical lever method, the measurement result is affected by the working distance of the angle sensor.

**Figure 3:**
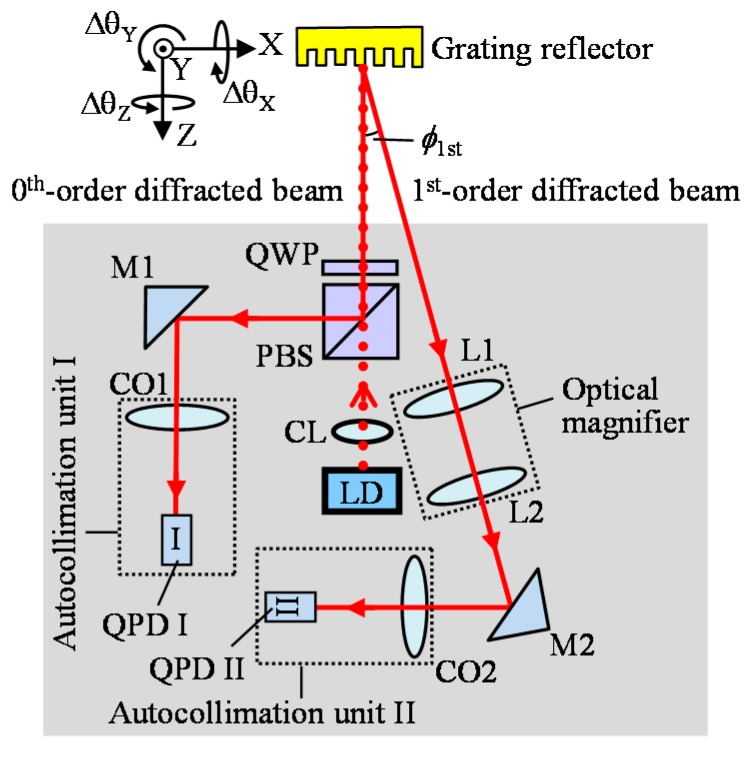
Three-axis autocollimator [52].

To address the aforementioned problems, the three-axis autocollimator has been proposed [52]. Figure 3 shows an optical setup of the three-axis autocollimator. The three-axis autocollimator is based on the conventional laser autocollimation [51] while utilizing a grating reflector for angle measurement. In the figure, diffracted beams whose orders are equal to or higher than two are not indicated for the sake of simplicity. The laser beam emitted from a laser diode (LD) is collimated by using a lens, and is projected onto the grating reflector. The zeroth-order diffracted beam and either the positive or negative first-order diffracted beam are focused on each of the photodiodes. In the three-axis autocollimator, the angular displacements Δ*θ*_X_ and Δ*θ*_Y_ of the grating reflector about the *X* and *Y* axes, respectively, can be described as follows [52]:

(5) $$\Delta \theta_{X}=\Delta x_{0}/K_{\theta X}$$

(6) $$\Delta \theta_{Y}=\Delta y_{0}/K_{\theta Y}$$

where *K_θX_* and *K_θY_*(=2*f*) are the conversion ratios of the angular displacements Δ*θ*_X_ and Δ*θ*_Y_ to the linear displacements Δ*x*_0_ and Δ*y*_0_ of the beam spot of the zeroth-order diffracted beam, respectively. As can be seen in the equations, the two-axis angular displacements Δ*θ*_X_ and Δ*θ*_Y_ can be detected by using the zeroth-order diffracted beam. Meanwhile, the zeroth-order diffracted beam is insensitive to the angular displacement Δ*θ*_Z_ of the grating reflector about the *Z*-axis. On the contrary, the first-order diffracted beam is sensitive against the angular displacements Δ*θ*_X_, Δ*θ*_Y,_ and Δ*θ*_Z_. Denoting the *X*-and *Y*-directional displacements of the first-order diffracted beam spot on the photodetector due to the angular displacements as Δ*x*_1_ and Δ*y*_1_, respectively, the following equation can be obtained [52]:

(7) $$\Delta x_{1}=K_{\theta X}\Delta \theta_{X}$$

(8) $$\Delta y_{1}=K_{\theta Y}\Delta \theta_{Y}+L\Delta \theta_{Z}$$

From Equations (6), (7) and (8), the following equation can be obtained:

(9) $$\Delta \theta_{Z}=\left(\Delta y_{1}-\Delta y_{0}\right)/L$$

As can be seen in the above equations, Δ*θ_Z_* can be detected by utilizing both the zeroth and the first-order diffracted beams.

**Figure 4:**
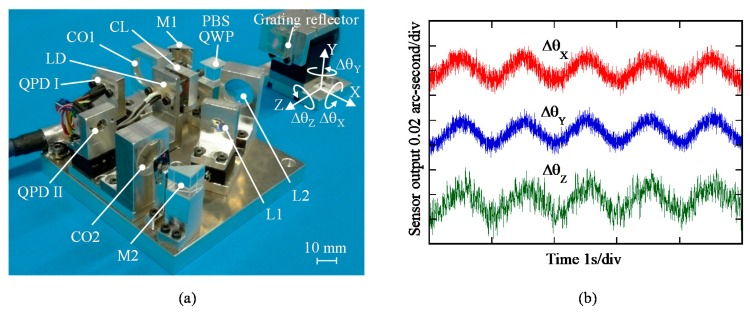
A photograph of the developed three-axis autocollimator, and the reading outputs from the optical sensor head of the three-axis autocollimator. (**a**) A photograph of the developed three-axis autocollimator. (**b**) Reading outputs from the optical sensor head when sinusoidal motion with a frequency of 1 Hz is given to each of the rotational axes [52].

A photograph of the optical sensor head for the three-axis autocollimator is shown in Figure 4a. By using the developed sensor head, the angular motion of a precision tilt stage about each of the axes with a small vibration amplitude was measured, while placing a grating reflector on the tilt stage table. Figure 4b shows the results. The experimental results have demonstrated that the developed three-axis autocollimator has the ability to distinguish 0.01 arc-second angular motion in all three axes [52]. It should be noted that the optical sensor head of the three-axis autocollimator can be designed in a compact size as same as that for a conventional linear encoder. By placing the optical sensor head of the three-axis autocollimator in the same manner as that of the conventional linear encoder, simultaneous measurement of three-axis angular displacement can be achieved over the wide travel range of a measurement target, while employing a single measurement laser beam [100]. It should be noted that the straightness/flatness error of the scale grating attached to the slide table could affect the measurement of angular error motions about the *X*-axis (roll) and the *Z*-axis (yaw), and are required to be compensated.

## 3. Multi-Axis Optical Displacement Sensors

### 3.1. Conventional Optical Sensors for Multi-Axis Displacement Measurement

**Figure 5:**
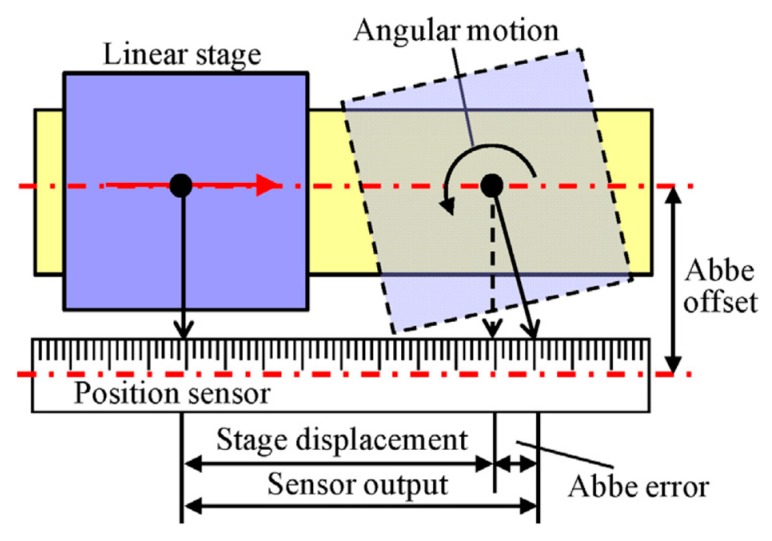
Influence of the Abbe error in position measurement of a linear slide table by a linear encoder whose scale is embedded to the side face of the slide table [4].

As described in the Introduction, multi-axis displacement measurement in the multi-axis Cartesian coordinate system can be carried out by employing multiple linear displacement sensors such as laser interferometers or optical linear encoders. In recent years, the optical linear encoders are expanding their applications [4,44]. Among several types of linear encoders, optical linear encoders employing a diffraction grating as the ruler for displacement measurement can realize a high resolution [28], and are suitable for the employment in precision positioning systems. In the optical linear encoder, sinusoidal interference signals, which are generated by superimposing the positive and negative diffracted beams obtained by projecting a light onto the diffraction grating, are employed to detect the relative displacement between the optical reading head and the diffraction grating. By interpolating the interference signals, a sub-nanometric resolution can be achieved [101,102,103]. Meanwhile, it is sometimes difficult, although not impossible [104,105], to align the measurement axis of a linear encoder with respect to the motion axis of a target of interest due to the mechanical configuration of a system in which the linear encoder is employed. In such a case, as can be seen in Figure 5 for example, an offset exists between the motion axis of the target of interest and the measurement axis of the linear encoder; this offset generates a certain amount of error in measurement of the target displacement (which is often referred to as the Abbe error [19]) when the target has angular error motions. This error can be compensated if the angular error motions of the target can be detected precisely [4].

It should be noted that a linear encoder cannot be applied for the applications such as a multi-axis planar motion stage where the distance between the reading head and the diffraction grating becomes large. In such applications, laser interferometers can be employed since they have a long measurement range and a long working distance along the laser axis [101,106]. By aligning the laser beam to comply with the Abbe principle, the influence of the Abbe error can be minimized [18,20,107,108]. Meanwhile, attention should be paid to stabilize the light wavelength of the laser beam since it is employed as the ruler for displacement measurement in the laser interferometers [109].

In principle, both a linear encoder and a laser interferometer are linear displacement sensors. For multi-axis displacement measurement, several scales and/or measurement laser beams are required to be employed. In such a case, attention should be paid to suppress the increase of measurement uncertainty due to the influence of the Abbe error at each axis as well as the influences of optical misalignments in a measurement system. Some novel positioning systems complying with the Abbe principle have been developed so far [18,20,107,108]; for example, by aligning the *X*-, *Y*-, and *Z*-directional measurement laser beams of interferometers to intersect at the measurement probe or the cutting tool, the Abbe error can be minimized [18,20,107,108]. However, in most of the cases, it is not so easy to design a system to comply with the Abbe principle due to the restrictions that come from the mechanical structure of the system. Although the influence of the Abbe error can be minimized by measuring and compensating the angular error motion of the target of interest [4], it is desired to measure the multi-axis displacement of a target of interest in a simple manner by using a single optical sensor.

### 3.2. Two-Axis Planar Encoder Using Two-Dimensional Scale Grating

To address the issues of conventional multi-axis displacement measurement systems for precision positioning, a two-axis planar encoder capable of measuring the two-axis displacement of a target of interest by using a single reading head has been developed [81]. By replacing the line scale grating in a conventional linear encoder to a planar scale grating and modify the optical reading head to read the diffracted beam in both the *X*- and *Y*-directions, two-axis in-plane displacement can be measured by using a single laser beam. A two-dimensional planar encoder having a nanometric resolution along the *X*- and *Y*-direction over an area of *ϕ*230 mm with the employment of a two-axis grating with a grating pitch of 4 μm has already been made commercially available [110], and have already been applied for semiconductor equipment [111,112]. Meanwhile, due to the limited working distance of the reading head in the planar encoders, most of their applications are limited to the positioning systems where the distance between the reading head and the planar scale grating is kept almost constant. In addition, a space for mounting the planar scale grating is required to be reserved in the positioning system; this restriction also limits the application of the planar encoder.

**Figure 6:**
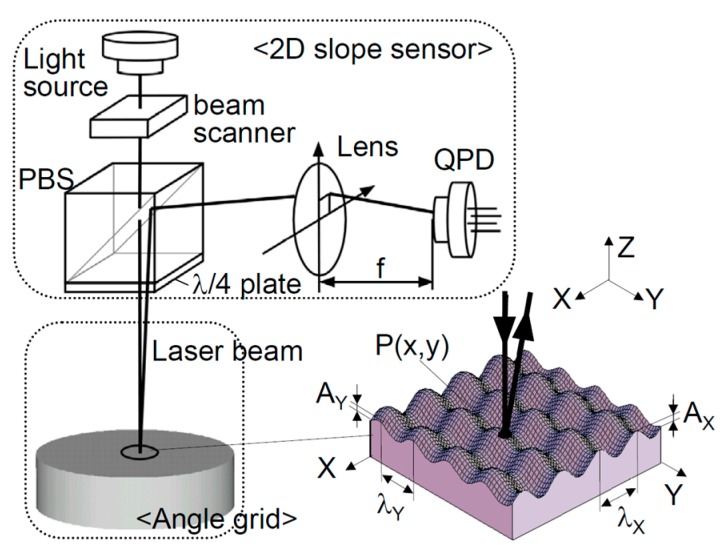
Two-axis planar encoder using two-dimensional scale grating [56].

Two-axis in-plane displacement can also be measured by employing an angle grid and an optical angle sensor based on the laser autocollimation [56,60,113]. In the method, as can be seen in Figure 6, two-axis in-plane displacement can be detected by reading the two-dimensional local slope of the two-dimensional sinusoidal pattern structures on the angle grid. The angle grid can be fabricated precisely by using the fast tool servo technique [80,114,115].

### 3.3. Three-Axis Planar Encoder Using Diffraction Scale Grating

#### 3.3.1. Principle of the Three-Axis Planar Encoder

**Figure 7:**
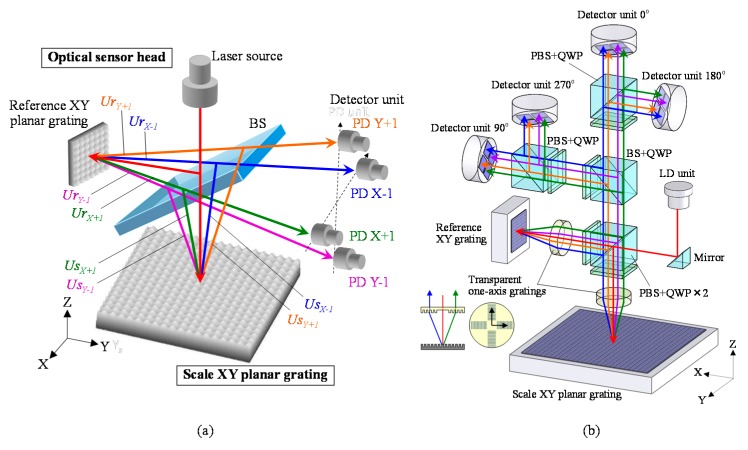
Three-axis planar encoder [58]. (**a**) A schematic of the principle of the three-axis planar encoder; (**b**) The detailed optical setup of the reading sensor head.

A simultaneous three-axis displacement measurement, which includes the *X*- and *Y*-directional in-plane displacement as well as the *Z*-directional out-of-plane displacement, with a single reading head can be realized by integrating the principle of the Michelson interferometer into that of the two-dimensional planar encoder with the planar scale grating [53,58]. Figure 7a shows a schematic of the principle of the three-axis planar encoder [58], in which the planar scale grating and the reference grating are employed. In the three-axis planar encoder, the reference grating is held stationary in the same manner as the conventional Michelson interferometer, while the planar scale grating can be moved in the *X*-, *Y*- and *Z*-directions. The employment of the reference grating enables the optical setup to measure the displacement of the planar scale grating along the Z-direction based on the principle of the conventional Michelson interferometer. By dividing a laser beam into two sub-beams by a beam splitter and project each of them onto the planar scale grating and the reference grating, diffracted beams along the positive and negative *X*- and *Y*-directions can be obtained. Figure 7b shows a schematic of the optical setup of the three-axis planar encoder. Positive/negative first-order diffracted beams from the planar scale grating and the reference grating can be superimposed by using the pair of transparent one-axis gratings as shown in the figure. This optical arrangement can generate the following four interference signals *I_X_*_+1_, *I_X_*_-1_, *I_Y_*_+1,_ and *I_Y_*_-1_ [58]:

(10) $$I_{X\pm 1}=\left(U_{s_{X\pm 1}}+U_{r_{X\pm 1}}\right)\;\left(\bar{U_{s_{X\pm 1}}+U_{r_{X\pm 1}}}\right)\;=2U_{0}{}^{2}\left[1+\cos\left\{\pm K\Delta x+v\left(1+\cos\theta \right)\Delta z\right\}\right]$$

(11) $$I_{Y\pm 1}=\left(U_{s_{Y\pm 1}}+U_{r_{Y\pm 1}}\right)\;\left(U_{s_{Y\pm 1}}+U_{r_{Y\pm 1}}\right)=2U_{0}{}^{2}\left[1+\cos\left\{\pm K\Delta y+v\left(1+\cos\theta \right)\Delta z\right\}\right]$$

where Δ*x*, Δ*y*, and Δ*z* are displacements of the planar scale grating along the *X*-, *Y*- and *Z*-directions, respectively, while *U*_0_ and *θ* represent the complex amplitude and the diffraction angle of the first-order diffracted beams, respectively. In the above equations, *k* = 2π/*g* and *v* = 2π/*λ*, where *g* is the grating pitch, and *λ* is the laser wavelength. By observing the interference signals and carrying out calculations, the signals *S*_Aα_, *S*_Bα_ (*α* = *X* ± 1, *Y* ± 1) described by the following equations can be obtained:

(12) $$S_{AX\pm 1}=\cos\left[\pm \left(2\pi /g\right)\Delta x+\left(2\pi /\lambda \right)\left(1+\cos\theta \right)\Delta z\right]$$

(13) $$S_{AY\pm 1}=\cos\left[\pm \left(2\pi /g\right)\Delta y+\left(2\pi /\lambda \right)\left(1+\cos\theta \right)\Delta z\right]$$

(14) $$S_{BX\pm 1}=\sin\left[\pm \left(2\pi /g\right)\Delta x+\left(2\pi /\lambda \right)\left(1+\cos\theta \right)\Delta z\right]$$

(15) $$S_{BY\pm 1}=\sin\left[\pm \left(2\pi /g\right)\Delta y+\left(2\pi /\lambda \right)\left(1+\cos\theta \right)\Delta z\right]$$

From the above equations, the three-dimensional displacements of the target of interest can be detected simultaneously by a single measurement laser beam.

**Figure 8:**
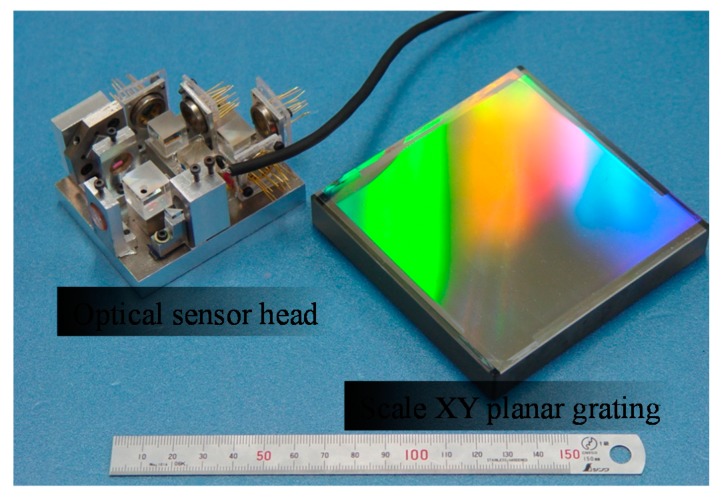
A photograph of the optical sensor head and the planar scale grating [58].

**Figure 9:**
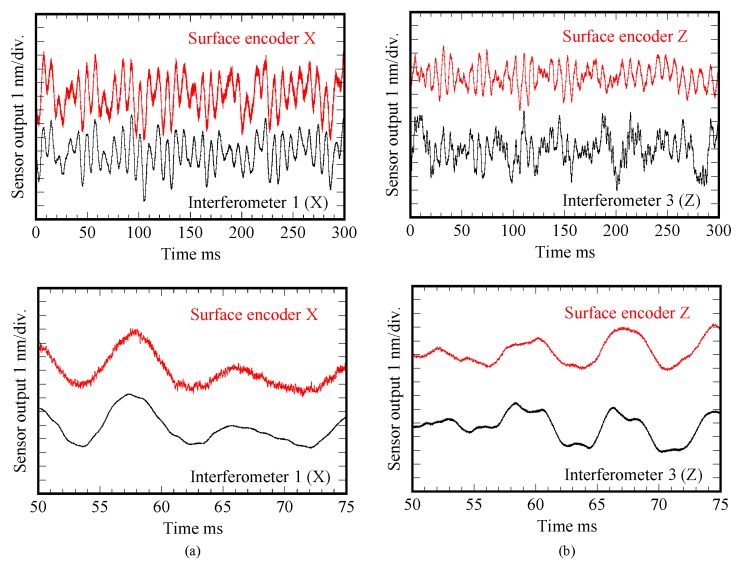
Nanometric dynamic motion of the scale grating measured by the three-axis planar encoder. (**a**) *X*-directional motion; (**b**) *Z*-directional motion [58].

Figure 8 shows a photograph of the developed prototype of the three-axis planar encoder [58]. Basic characteristics of the developed three-axis planar encoder were verified in experiments. In the experiments, the planar scale grating was held stationary, while measuring the small translational displacement of the planar scale grating along the *X*-, *Y*- and *Z*-directions by the optical sensor head of the three-axis planar encoder. In the experiment, a commercially available laser interferometer was also employed as the reference sensor. Figure 9 shows the result. The experimental results have demonstrated that a resolution of better than 1 nm in all the three axes can be achieved by employing the planar scale grating having a grating pitch of smaller than 1 μm [58]. In the three-axis planar encoder described above, the positive and negative first-order diffracted beams from the scale grating are employed to detect the three degree-of-freedom displacement of a target of interest. It should be noted that, by employing the zeroth-order diffracted beam for measurement of the out-of-plane (*Z*) displacement, simultaneous measurement of the three-directional translational displacements can also be carried out [116].

**Figure 10:**
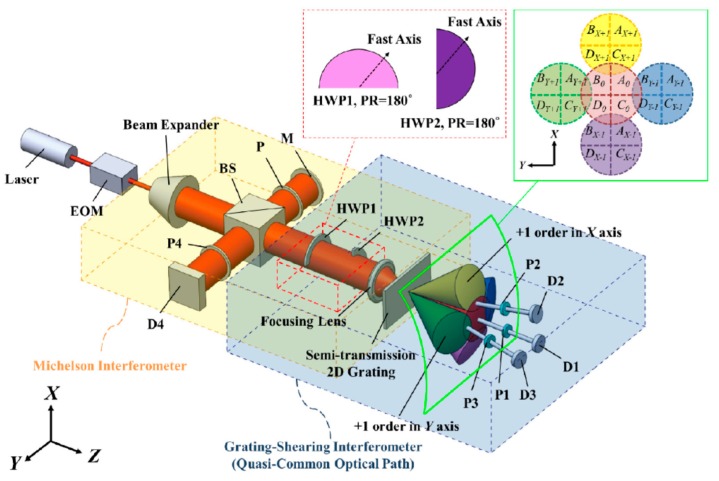
System configuration for 3-DOF displacement measurement with the semi-transmission 2D grating [78].

In the above three-axis planar encoder, the *X*- and *Y*-directional positive and negative first-order diffracted beams from the reflective-type planar scale grating and the reference grating are employed to detect three-axis displacement. The three-DOF displacement measurement can also be carried out by employing a semi-transmission 2D grating. Figure 10 shows an optical setup for three-DOF displacement measurement with the semi-transmission 2D grating reflector [78]. The optical setup is designed in such a way that the focused laser beam is projected onto the grating so that the interference signals between the transparent zeroth-order diffracted beam and each of the first-order diffracted beams can be obtained. By detecting the phase difference between the transparent zeroth-order diffracted beam and the interference signals, in-plane displacement (along the *X*- and *Y*-directions) can be obtained. In the optical setup, the axial displacement of the 2D grating (along the *Z*-direction) is detected based on the principle of conventional Michelson interferometer. This setup can also detect the three-dimensional displacements of the target of interest simultaneously by a single measurement laser beam.

#### 3.3.2. Multi-Beam Planar Encoder with a Mosaic Scale Grating

In the semiconductor industry, regarding a wafer size, a travel range of over 500 mm in each of the in-plane directions (*X*- and *Y*-directions) is required for a positioning system. Position sensors to be employed in such a positioning system are thus required to have a measurement range enough to cover the long stroke of the positioning system. The measurement range of the three-axis planar encoder along the in-plane axes depends on the size of the planar scale grating. A size of the planar scale grating is therefore required to be larger than 500 mm × 500 mm to fulfill the requirement from the semiconductor industry [117]. However, it is not so easy to fabricate such a large-area planar scale grating in laboratories and/or institutes where fabrication facility and research budget are limited. Furthermore, the gravitational deformation of the planar scale grating could degrade the accuracy of the three-axis planar encoder.

**Figure 11:**
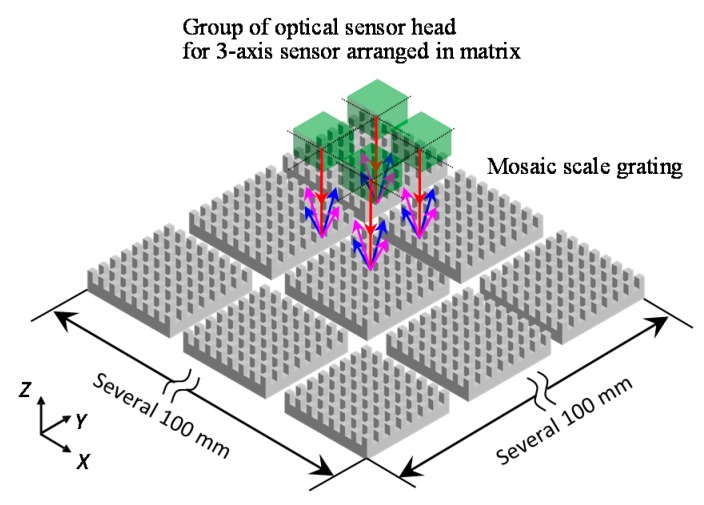
Four-probe three-axis planar encoder with a 2D mosaic scale grating [61].

To overcome the aforementioned issues on the large-area planar scale grating, a concept of the four-probe optical sensor head for the three-axis planar encoder with a mosaic scale grating, a schematic of which is shown in Figure 11, has been proposed [61]. In the proposed method, with the enhancement of the idea of mosaic gratings [118,119], a group of small planar scale gratings arranged in a matrix is read by an optical sensor head having multiple measurement laser probes so that the mosaic scale grating can be treated as an “effective” large-area planar scale grating. The small planar scale grating is easier to fabricate compared with the large-area planar scale grating. In addition, fabrication costs can also be reduced. Furthermore, the mosaic scale grating can reduce the influence of the gravitational deformation of the large-area planar scale grating.

**Figure 12:**
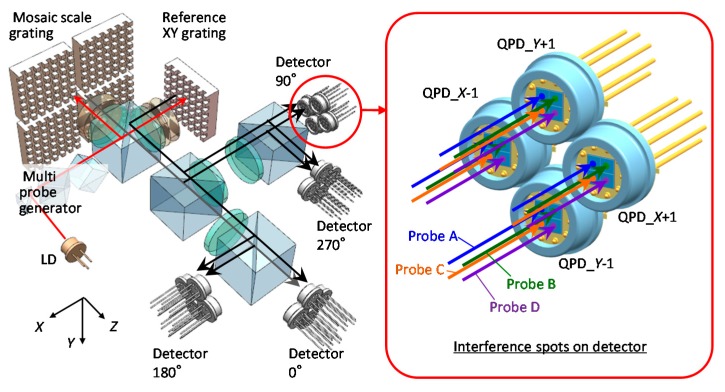
The optical setup of the four-probe three-axis planar encoder with a mosaic scale grating [61].

A schematic of the three-axis planar encoder with a mosaic scale grating is shown in Figure 12. A certain gap (width: *g*_Scale_) exists between neighboring two small planar scale gratings in the mosaic scale grating. When a measurement laser beam from the optical sensor head is projected onto the gap, the accurate displacement measurement cannot be carried out. Meanwhile, this problem can be overcome by employing a reading sensor head having multiple sub-beams that are arranged to have a distance *d*_Beam_ larger than *g*_Scale_. With the arrangement, at least one of the sub-beams can detect the three-axis displacement of the mosaic scale grating [61]. The sub-beams can be generated by using conventional optical components; for example, four sub-beams can be generated by dividing a single measurement laser beam with a pair of cube beam splitters. In the case of an optical sensor head having four sub-beams aligned in a matrix of 2 × 2, since four first-order diffracted beams (*X*-positive, *X*-negative, *Y*-positive and *Y*-negative) will be generated by each sub-beam projected onto the mosaic scale grating, totally 16 interference signals are required to be observed in the optical sensor head of the three-axis planar encoder. It should be noted that the optical sensor head can be designed in a simple manner by employing quadrant photodiodes (QPDs) as the photodetectors as shown in Figure 12. In the optical sensor head, each of the sub-beams acts as an independent measurement beam, and can detect three-axis displacement of the mosaic scale grating.

**Figure 13:**
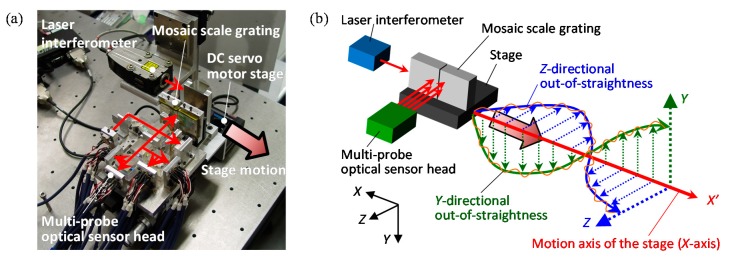
Evaluation of the out-of-straightness motion error of the linear slide by using the four-probe three-axis planar encoder with a 2D mosaic scale grating [61]. (**a**) A picture of the setup; (**b**) A schematic of the setup.

**Figure 14:**
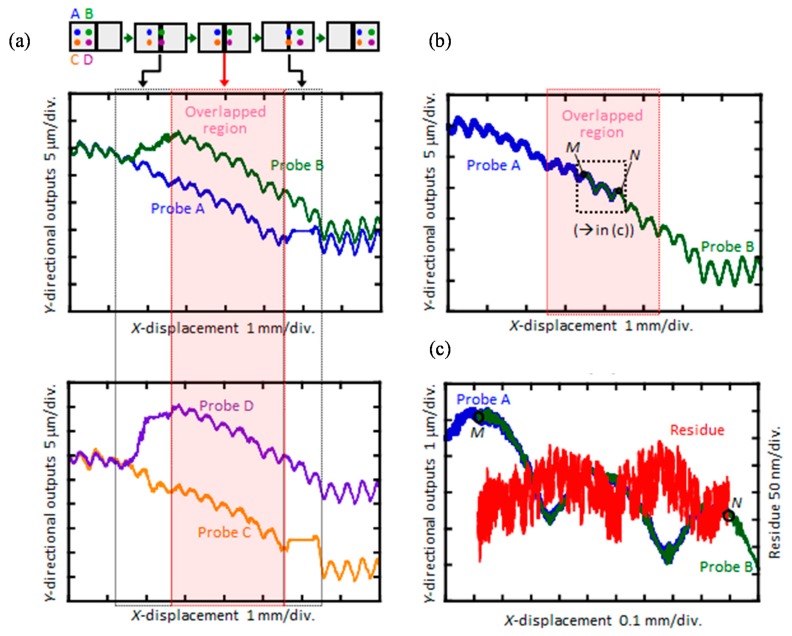
Out-of-straightness motion error of the linear slide evaluated by the four-probe three-axis planar encoder with a 2D mosaic scale grating [61]. (**a**) *Y*-directional out-of-straightness; (**b**) The stitched *Y*-directional out-of-straightness; (**c**) Residue of the stitching.

Feasibility of the three-axis planar encoder having sub-beams and a mosaic scale grating has been verified through some experiments by using the setup shown in Figure 13a. In the experiments, as can be seen in Figure 13b, a mosaic scale grating was mounted on the moving table of a DC servo motor stage, and the relative motion of the moving table along the *X*-direction in the figure was detected by the four sub-beams (Beams-A, -B, -C and –D) in the optical reading head. It should be noted that a commercially available laser interferometric displacement sensor was employed as a reference sensor. Figure 14a–c show the *Y*-displacement (corresponding to the out-of-straightness) of the moving table measured by each of the beams. In the figure, the region plotted in a dashed line corresponds to the output obtained at the scale gap. As can be seen in the figure, Beam-A(C) could successfully detect the scale *Y*-position while Beam-B(D) could not detect the scale *Y*-position correctly while it was on the scale gap. By stitching the outputs of Beams-A(C) and –B(D), the influence of the scale gap could successfully be avoided as shown in Figure 14b,c. These results have demonstrated the feasibility of the proposed four-probe optical sensor head for the three-axis planar encoder with the mosaic scale grating.

It should be noted that the measurement sensitivity of each sub-beam can be compensated by using a sub-beam arbitrarily chosen from the four sub-beams as a reference probe, and carry out matrix calculations [61] while measuring the same small planar scale grating. In the same manner, the misalignment of each of the small planar scale gratings arranged in a matrix can be compensated. Meanwhile, one of the disadvantages of this method is that the measurement uncertainty could increase due to the error propagation, and the issue is required to be addressed for measurement of long-range in-plane displacements.

#### 3.3.3. A Six-Degree-Of-Freedom Planar Encoder

Both the three-axis autocollimator and the three-axis planar encoder employ a planar scale grating as their target reflector. Therefore, by combining the functions of the three-axis autocollimator and the three-axis planar encoder in the same optical sensor head, a six-degree-of-freedom (DOF) planar encoder capable of simultaneously measuring the three-axis translational displacement and the three-axis angular displacement can be realized [54]. Since the optical sensor head has only a single measurement laser beam, the six-DOF planar encoder is expected to be employed as a compact multi-axis displacement sensor for precision positioning.

**Figure 15:**
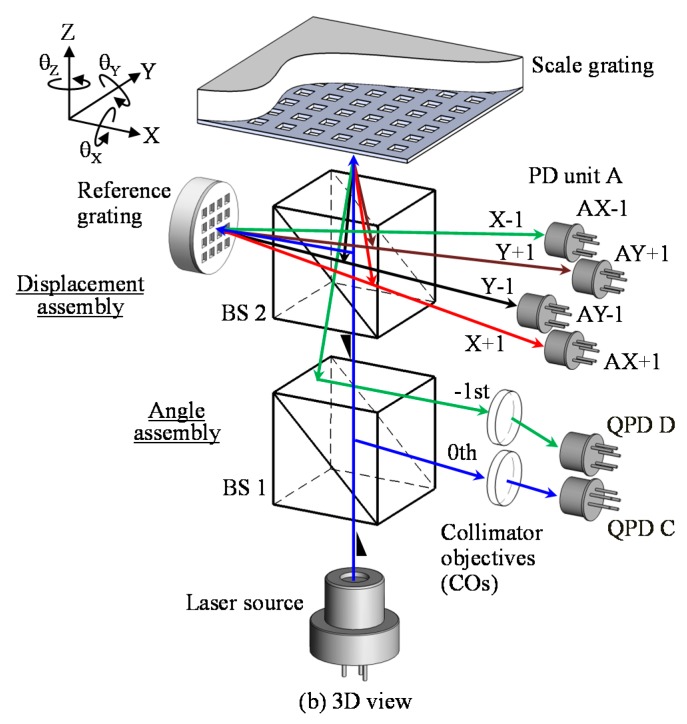
A six-degree-of-freedom planar encoder [54].

A schematic of the six-DOF planar encoder is shown in Figure 15 [54]. The optical sensor head of the six-DOF planar encoder is mainly composed of a displacement assembly and an angle assembly for the three-axis displacement measurement and the three-axis angle measurement, respectively. The displacement assembly has a reference grating, a polarizing beam splitter (PBS) and a PD unit composed of four QPDs, while the angle assembly has a beam splitter (BS), collimator objective (CO) and two QPDs (QPD-C and QPD-D). As can be seen in the figure, the displacement assembly and the angle assembly share the collimated laser beam from a laser diode (LD) as the measurement laser beam, as well as the planar scale grating. This optical configuration enables the six-DOF planar encoder to carry out three-axis displacement measurement and three-axis angle measurement simultaneously at the same point on the planar scale grating.

**Figure 16:**
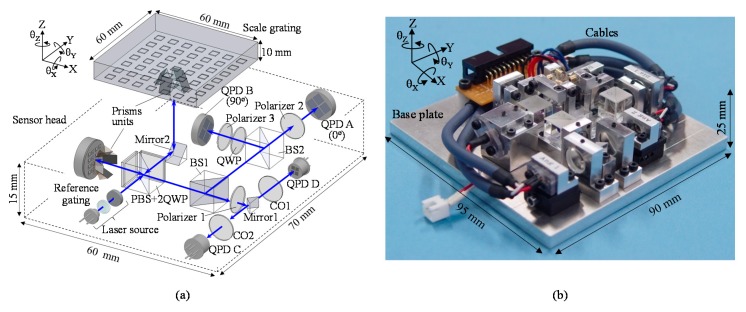
The optical setup of the planar encoder [54]. (**a**) A schematic of the reading head in the six-degree-of-freedom planar encoder; (**b**) A photograph of the reading head.

Figure 16a shows the optical setu for the six-DOF planar encoder designed for the planarmotion stage system [54,55]. In the displacement assembly, the three-axis displacement of the planar scale grating is measured based on the principle of the three-axis planar encoder [53]. The measurement laser beam emitted from LD is divided into two beams by PBS, and the divided beams are projected onto the scale grating and the reference grating after passing through a quarter-wave plate (QWP) to generate positive and negative first-order diffracted beams. 

As can be seen in the figure, a prism unit is employed to make the diffracted beams from the grating to be parallel with the projected beam. The diffracted beams pass through QWP again, and are superimposed at the PBS to generate interference signals. The generated interference signals are detected by the four QPDs. Meanwhile, in the angle assembly, the three-axis angular displacement of the scale grating is measured based on the principle of the three-axis autocollimator [52]. As can be seen in the figure, the zeroth-order diffracted beam from the scale grating is focused on the QPD-C, while the first-order diffracted beam is focused on the QPD-D. Figure 16b shows a photograph of the developed optical sensor head of the six-DOF planar encoder [54].

**Figure 17:**
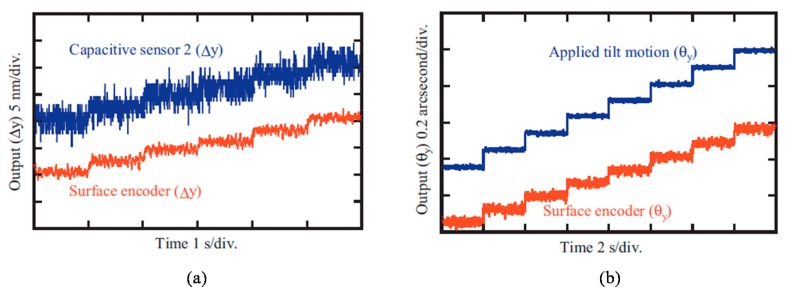
Translational motion and angular motion of the scale grating measured by the six-DOF planar encoder [54]. (**a**) *Y*-directional translational displacement; (**b**) Angular displacement about the *Y*-axis.

Experiments were carried out to verify the feasibility of the developed six-DOF planar encoder. In the experiments, the optical sensor head was held stationary, while the scale grating mounted on a multi-axis piezoelectric (PZT) stage system to give translational displacement along the three axes and angular displacement about the three axes. Figure 17a,b show the *Y*-directional translational displacement and the angular displacement about the *Y*-axis, respectively, detected by the six-DOF planar encoder. It should be noted that all the displacements can be measured simultaneously. As can be seen in the figure, a resolution of 2 nm for the measurement of in-plane and out-of-plane translational displacements was achieved. Furthermore, resolutions of 0.1 arc-second, 0.1 arc-second, and 0.3 arc-second for measurement of the angular displacements about the *X*-, *Y*- and *Z*-axes, respectively, were achieved. It should be noted crosstalk between the displacement assembly and the angle assembly could occur since they share the first-order diffracted beams in the optical sensor head to detect translational displacements and angular displacements [54]. Meanwhile, the influence of the crosstalk can be minimized by optimizing the arrangement of the assemblies and each of the optical components in the optical sensor head [55].

## 4. Fabrication and Verification of Scale Grating

### 4.1. Fast-Tool-Servo Cutting of Angular Scale Gratings

Multi-axis planar encoders employ two-dimensional pattern structures on a planar scale grating as rulers for measurement of the three-axis translational displacement and/or the three-axis angular displacement. The pattern structures are required to be fabricated over a large area, since the in-plane measurement range of the planar encoder depends on the size of the planar scale grating. In most of the precision measuring instruments such as a micro coordinate measuring machine (CMM) or a scanning white-light interferometer, a measurement range of 100 mm along one axis is enough. In the case of positioning systems employed in the semiconductor industry, a measurement range of over 450 mm is required; however, as described in the previous section, a mosaic scale grating composed of several small scale gratings (for example, the planar scale grating having a size of 100 mm × 100 mm) can be employed as the large scale grating, while avoiding the increase of fabrication cost and reducing the influence of the gravitational deformation of the large scale grating [61].

**Figure 18:**
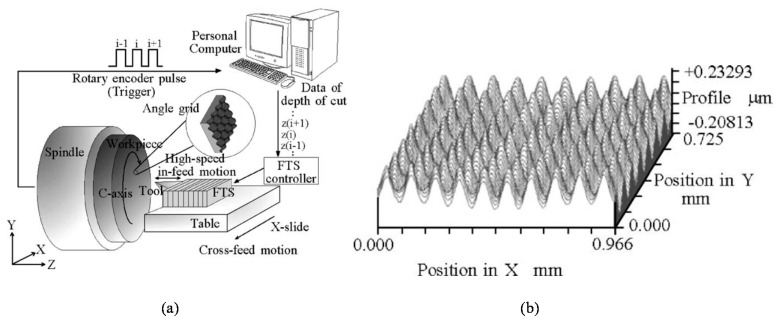
Fabrication of an angular scale grating by using fast tool servo (FTS) technique on an ultra-precision lathe [80]. (**a**) A schematic of the setup; (**b**) Profile of the fabricated two-dimensional sinusoidal angle grid.

Traditional line-pattern scale grating can be fabricated by ruling engines [120,121,122], in which a single-point diamond cutting tool is employed to cut the line pattern structures. With the enhancement of a high-precision positioning system, a highly-accurate line pattern structures can be fabricated over a large area. Meanwhile, it is difficult to apply the traditional ruling engines to fabricate planar scale gratings. This issue can be addressed by employing the fast tool servo (FTS) technique [80,81,113,114,115]. Figure 18 shows a schematic of the FTS unit developed for the fabrication of two-dimensional sinusoidal pattern structures. The FTS unit is mainly composed of a single-point diamond cutting tool, a precision PZT actuator that enables the FTS unit to generate fast in-feed motion of the cutting tool, and a precision displacement sensor for the detection of tool motion. By mounting the FTS unit onto an ultra-precision lathe, complicated three-dimensional profiles such as the two-dimensional sinusoidal angle grid with a pattern pitch of 100 μm [80] can be fabricated. With the employment of a diamond cutting tool having a further smaller nose radius, the 2D angle grid with a pattern pitch of smaller than 10 mm can be fabricated over a large area [81].

Meanwhile, it takes a long time for the fabrication of large-area scale grating, and the degradation of fabrication accuracy due to the tool wear cannot be avoided. In addition, tool marks on the fabricated grating surface could generate unnecessary ghost diffraction or stray light. Furthermore, the minimum pattern pitch that can be realized by the diamond cutting is limited by the tip radius of the cutting tool [81].

### 4.2. Interference Lithography of Diffraction Scale Gratings

For the multi-axis planar encoder employing diffracted beams from the planar scale grating, the pitch of the pattern structures on the grating is designed to be in the scale of micrometers [110] or sub-micrometer [54], while symmetric pattern structures having uniform amplitude over the entire scale grating surface are preferred to obtain high diffraction efficiency along the *X*- and *Y*-directions. Such fine pattern structures can be fabricated through the photolithography process. In the photolithography process, grating pattern structures are fabricated by transferring the mask pattern onto the grating substrate. By repeating the pattern exposure with the help of high-precision positioning technology, fine grating pattern structures having micrometric or sub-micrometric pitch can be fabricated over a wide area [123]. Meanwhile, the photolithography process requires expensive semiconductor equipment and photo-masks, which make the grating fabrication expensive. On the contrary, the laser interference lithography [124], in which interference fringe patterns generated by superimposing several laser beams are transferred to the grating substrate, can fabricate grating pattern structures having a micrometric or sub-micrometric pitch without using expensive photo-masks and expensive equipment. Therefore, the laser interference lithography is suitable to fabricate planar scale gratings in small companies or laboratories, where research budgets and fabrication facilities are limited.

**Figure 19:**
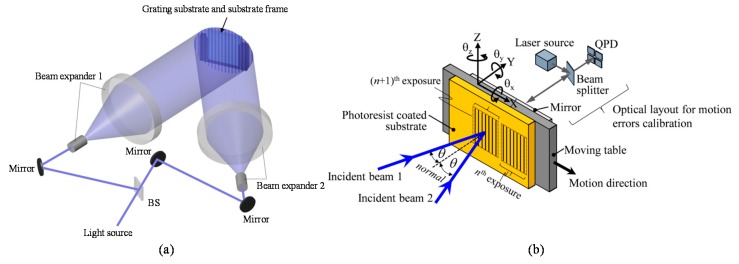
Optical setups for interference lithography. (**a**) An example of the division of amplitude systems [59]; (**b**) Compensation of angular motion error by the optical angle sensor [129].

There are two major types of optical configuration for the laser interference lithography: the one is referred to as the division of amplitude system [59,125,126,127,128,129]. Figure 19a shows an optical setup of the phase-shift laser interference lithography [59], which is a kind of division of amplitude system. As can be seen in the figure, a laser beam is at first divided into two sub-beams by a beam splitter. After that, the sub-beams are projected onto a substrate, while the angle of incidence of each sub-beam is adjusted by a mirror. The optical path difference between the two sub-beams will generate line interference fringe patterns on the substrate, and the fringe patterns will be transferred to the resist layer prepared on the substrate surface. This optical setup can realize the fabrication of large-area diffraction grating by expanding the sub-beams by using beam expanders. In addition, the grating pitch can be adjusted by controlling the angles of incidence of the sub-beams with respect to the grating substrate surface. Due to the principle of interference fringe generation, the Y-directional misalignment of the substrate will not affect the grating pitch. By scanning the substrate along the X-direction with the fringe locking system, line pattern structures can be exposed over a long travel range [124]. Meanwhile, the optical setup based on the two-beam interference lithography tends to become complicated and weak against external disturbances due to the long optical paths of the sub-beams. Some methods have been proposed so far to overcome the problem [127,128,129]. For example, the angular motion errors *θ_X_* and *θ_Z_* of the substrate about the *X* and *Z*-axes, respectively, will affect the profile uniformity of the pattern structures as well as the grating pitch. The influences of these angular motion errors can be compensated by using the optical angle sensor as shown in Figure 19b [129]. Furthermore, by fabricating the array of one-axis grating as shown in the figure and preparing an optical sensor head with a pair of optical probes based on the concept of mosaic scale grating, the pattern exposure system can be simplified without the complicated fringe locking system [129].

**Figure 20:**
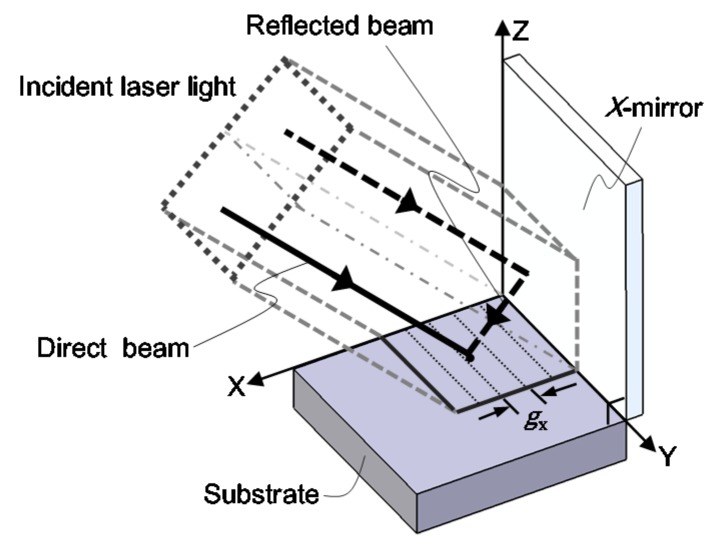
Optical setups for interference lithography based on the division of wavefront systems [82].

Another major type of optical configuration for the laser interference lithography is the division of wavefront system [82,130,131,132,133,134,135,136]. Figure 20 shows an optical setup of a Lloyd’s mirror interferometer, which is a kind of the division of wavefront systems [82]. The Lloyd’s mirror interferometer composed of a substrate and a mirror, which is placed at the right angle with respect to the substrate. As can be seen in the figure, a part of the beam directly projected onto the substrate (direct beam) will be superimposed by the other part of the beam reflected by the mirror (reflected beam) to generate line interference fringe patterns on the substrate surface. The direct beam shares most of the optical path in the simple optical setup with the reflected beam: this feature allows the Lloyd’s mirror interferometer to have superior stability against external disturbances. Furthermore, by employing an additional mirror to the conventional one-axis Lloyd’s mirror, two-dimensional pattern structures can be fabricated at a single pattern exposure process [82,130,131,132] over a wide substrate surface. In the following of this section, fabrication of a large-area planar scale grating by a non-orthogonal two-axis Lloyd’s mirror interferometer is presented.

**Figure 21:**
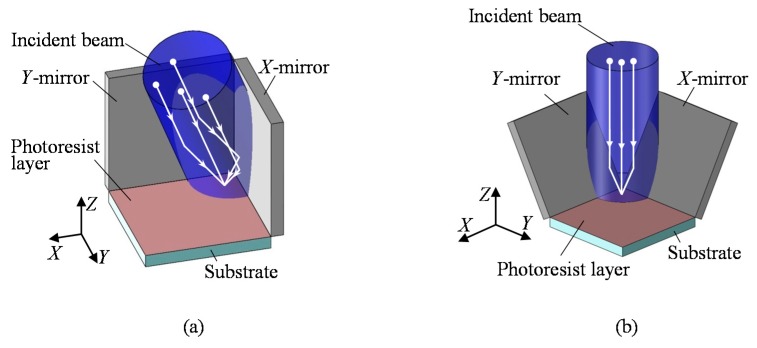
Two-axis Lloyd’s mirror interferometer [83]. (**a**) Orthogonal type; (**b**) Non-orthogonal type.

The conventional one-axis Lloyd’s mirror interferometer can fabricate line pattern structures at a single exposure process. For the fabrication of two-dimensional pattern structures, additional exposure process after rotating the substrate 90 degrees about the normal of the substrate is required. However, this two-step exposure process makes the amplitudes of pattern structures in the two orthogonal directions different, resulting in the difference of the diffraction efficiencies in the two axes [133]. This problem can easily be solved by employing an additional mirror to construct an orthogonal two-axis Lloyd’s mirror interferometer unit [87,130,131] as shown in Figure 21a. However, for the fabrication of a large-area planar scale grating, a large interferometer unit will be required; it is not so easy to construct such a large interferometer unit. In addition, severe optical alignments are required for the pair of mirrors and the angle of incidence of the laser beam with respect to the interferometer unit [132], while perfect polarization modulation control is difficult [134]. On the contrary, the optical configuration of the non-orthogonal two-axis Lloyd’s mirror interferometer, in which a pair of mirrors are placed to have angles of (90 + α) degrees with respect to the substrate surface as shown in Figure 21b, allows the interferometer unit to be designed in a compact size and to be robust against optical misalignments of the optical components and the laser beam projected onto the interferometer [82,83,84,85].

**Figure 22:**
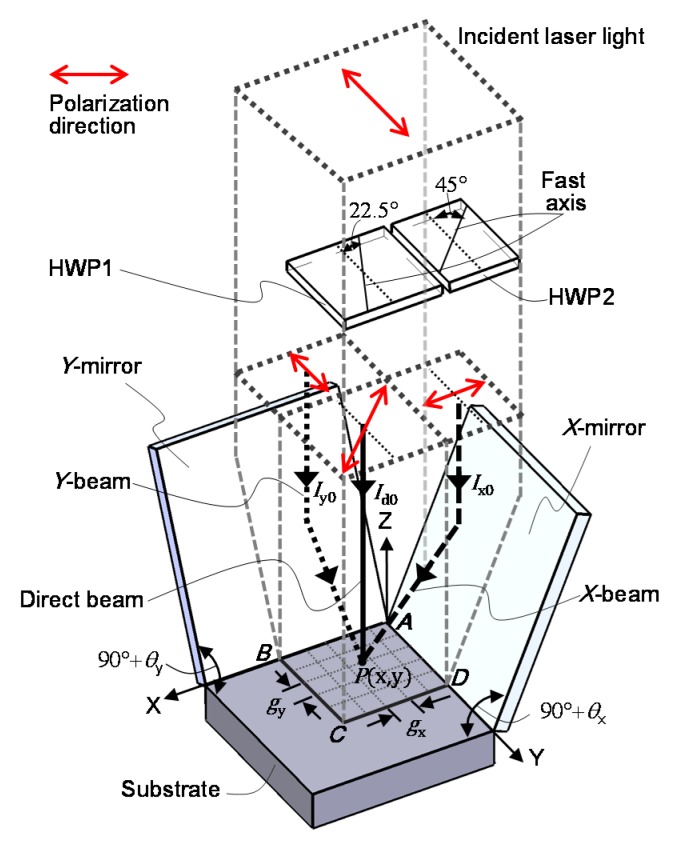
An optical setup for the non-orthogonal two-axis Lloyd’s mirror interferometer [82].

A schematic of the optical setup for the non-orthogonal two-axis Lloyd’s mirror interferometer is shown in Figure 22 [82]. In the setup, a laser beam is projected onto the substrate with a right angle. On the substrate surface, two-axis interference fringe patterns are generated as the consequence of the superposition of the direct beam, which is directly projected onto the substrate surface, and the reflected beams, which are projected onto the substrate surface after being reflected by the *X*- or *Y*-mirror. Now the complex amplitude of the laser beam projected onto the substrate surface can be described by the following equation [82]:

(16) $${\vec{E}}_{n}\left(\vec{r}\right)=E_{n}{\vec{e}}_{n}\exp\left(i{\vec{k}}_{n}\cdot \vec{r}+\phi_{n}\right)$$

where $\vec{r}$, ${\vec{E}}_{n}$, ${\vec{e}}_{n}$ and $\phi_{n}$ are the position vector, electric field amplitude, unit vector of the polarization direction of the laser beam and the initial phase. By denoting the wave vectors of the direct beam, the beam reflected by the *X*-mirror (*X*-beam) and that reflected by the *Y*-mirror (*Y*-beam) as ${\vec{k}}_{1}$, ${\vec{k}}_{2}$ and ${\vec{k}}_{3}$, respectively, the interference fringe patterns to be generated on the substrate surface can be described by the following equation [82]:

(17) $$I\left(\vec{r}\right)=\sum_{l=1}^{3}E_{l}^{2}+2\sum_{l=2}^{3}\sum_{k<l}E_{k}E_{l}{\vec{e}}_{k}\cdot {\vec{e}}_{l}\cos\left\{\left({\vec{k}}_{k}-{\vec{k}}_{l}\right)\cdot \vec{r}+\phi_{k}-\phi_{l}\right\}$$

where ${\vec{k}}_{1}$, ${\vec{k}}_{2}$ and ${\vec{k}}_{3}$ can be expressed as follows:

(18) $${\vec{k}}_{1}=\frac{2\pi }{\lambda }\left(\begin{array}{c} 0\\ 0\\ -1 \end{array}\right),\;{\vec{k}}_{2}=\frac{2\pi }{\lambda }\left(\begin{array}{c} \sin2\theta_{1Y}\\ 0\\ -\cos2\theta_{1Y} \end{array}\right),\;{\vec{k}}_{3}=\frac{\sqrt{2}\pi }{\lambda }\left(\begin{array}{c} 0\\ \sin2\theta_{2X}\\ -\cos2\theta_{2X} \end{array}\right)$$

In the above equations, *λ* is the light wavelength of the laser beam, while *θ_Y_* and *θ_X_* are the angles of tangent vectors of the *X*- and *Y*-mirrors, respectively, with respect to the normal of the substrate. From the above equations, the pitches of the interference fringe *g_X_* and *g_Y_* along the *X*- and *Y*-directions, respectively, can be obtained as follows [82]:

(19) $$g_{X}=\frac{\lambda }{\sin2\theta_{y}},g_{Y}=\frac{\lambda }{\sin2\theta_{x}}$$

It should be noted that the interference fringe pattern component as the consequence of the interference between the *X*-beam and *Y*-beam is not necessary for the fabrication of the two-dimensional (2D) pattern structures for the planar scale grating; the influence of this component can effectively be reduced by applying the polarization modulation control technique [82].

**Figure 23:**
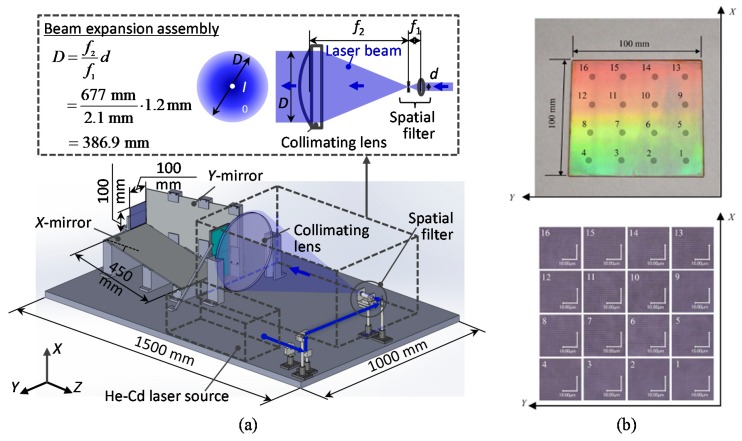
The non-orthogonal two-axis Lloyd’s mirror interferometer for the fabrication of large-area two-dimensional scale gratings [83]. (**a**) Optical setup; (**b**) A photograph and microscopic images of the fabricated large-area two-dimensional scale grating.

Figure 23a shows a schematic of the non-orthogonal two-axis Lloyd’s mirror interferometer designed for the fabrication of large-area planar scale grating [83]. A laser beam from a He-Cd laser source is at first collimated by a collimating lens. The collimated laser beam is then expanded by the beam-expansion unit, which is composed of a beam expander based on the Keplerian configuration.

The collimated laser beam with a large diameter is projected onto the interferometer unit, which is composed of the *X*- and *Y*-mirrors and a substrate with a photoresist layer. To eliminate the interference fringe component generated by the interference between the *X*- and *Y*-beams, the polarization modulation control unit composed of two half-wave plates is employed. Figure 23b shows an example of the large-area planar scale gratings fabricated by the setup shown in Figure 23a. As can be seen in the figure, grating pattern structures were successfully fabricated over an area of 100 mm × 100 mm on the substrate surface.

**Figure 24:**
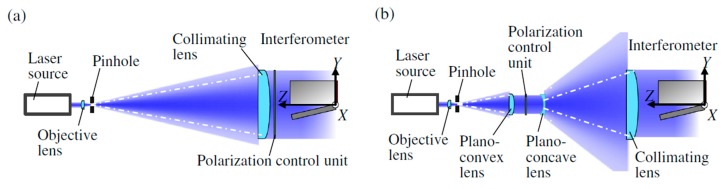
Beam expansion unit in the non-orthogonal two-axis Lloyd’s mirror interferometer [84]. (**a**) the conventional beam expansion assembly; (**b**) the modified beam expansion assembly.

It should be noted that the optical setup shown in Figure 23a requires large-scale half-wave plates whose sizes are comparable to the size of the substrate to be exposed by the interference fringe patterns. In general, such a large-scale half-wave plate with small retardation is difficult to prepare, and hence the polarization modulation control becomes imperfect, resulting in the distortion of the fabricated 2D pattern structures. This issue can be addressed by modifying the beam expansion assembly as shown in Figure 24 [84]. In the modified beam expansion assembly, the beam diameter is at first adjusted so that it would fit the size of half-wave plates with small retardation. After that, the beam is magnified by the beam expander designed in the Galilean configuration. Furthermore, homogenization of the light intensity in the collimated laser beam with the employment of a beam shaper [135] could reduce the amplitude deviation of the fabricated pattern structures on planar scale gratings [85].

### 4.3. Evaluation of Pitch Deviations of a Two-Dimensional Scale Grating

The accuracy of the grating pitch of a planar scale grating is important since the planar encoders utilize the grating pitch as the ruler for measurement of the displacement of a target of interest. As can be seen in Equations (13)–(16), deviation of the grating pitch Δ*g* directly affects the interference signals in the planar encoder, resulting in the nonlinear error components in the measurement of in-plane displacements. In addition, out-of-flatness of the planar scale grating would affect the measurement of the out-of-plane displacement, and would result in the nonlinear error component in the measured out-of-plane displacement. Therefore, it is important to evaluate the pitch deviation and the out-of-flatness of the planar scale grating and compensate for their influences on the displacement measurement in the planar encoders.

A pitch deviation of the planar scale grating can be evaluated by using critical-dimension atomic force microscopes (CD-AFMs) [137,138,139]. Since the CD-AFMs can evaluate the profile of each pattern structure on the grating, pitch deviation can be obtained as well as the absolute grating pitch. Meanwhile, the measurement throughput of the CD-AFM is not so high. Although some efforts have been made to expand the measurable area of the CD-AFMs [140], in most of the cases, the measurable area is limited to be several-hundred μm by several-hundred μm. Therefore, it is not a realistic way to apply the CD-AFMs for the evaluation of large-area planar scale gratings. For such a purpose, optical diffractometers can be employed; by detecting the diffraction angle of the diffracted beams from the scale grating in the Littrow configurations, the grating pitch can be obtained based on the grating equation [141,142]. Another candidate for the evaluation of planar scale gratings is the linear scale comparator [143,144,145]. In the linear scale comparator, an edge of each grating pattern structure is detected by using focused light, while the displacement of the grating pattern structure is measured by using a laser interferometer. Therefore, the linear scale comparator can be employed to evaluate the grating pitch deviation as well as the absolute pitch. On the other hand, since the focused light is employed to detect the edges of each grating pattern structure, it becomes more difficult to carry out the edge detection due to the light diffraction limit when the grating pitch is reduced to be less than a few micrometers. Furthermore, to avoid the influences of external disturbances on displacement measurement by the laser interferometer, severe environmental control is required; this prevents the linear scale comparators to be employed in general-purpose laboratory conditions. Furthermore, it is not so easy to modify the mechanical structure of linear scale comparator for the evaluation of planar scale gratings from the viewpoint of cost and the required experimental facilities. It should also be noted that both the CD-AFMs and the linear scale comparators cannot evaluate the out-of-flatness of the target grating.

**Figure 25:**
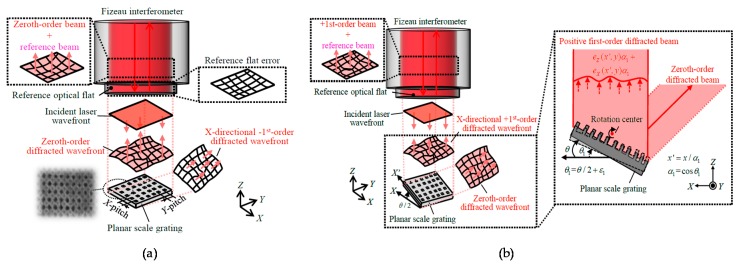
Evaluation of the pitch deviation of large-area planar scale gratings based on the Fizeau interferometer [88]. (**a**) Setup for measurement of zeroth-order diffracted beam wavefront; (**b**) Setup for measurement of the positive first-order diffracted beam wavefront.

In responding to the background described above, for the evaluation of the pitch deviation of large-area planar scale gratings, a method based on the Fizeau interferometer has been proposed [86,87,88]. In the proposed method, not only the zeroth-order diffracted beam but also the positive and negative first-order diffracted beams from the scale grating are obtained by placing a planar scale grating under inspection in the Littrow configuration. By using the wavefront information in the obtained beams, the *X*- and *Y*-directional pitch deviations and the out-of-flatness of the planar scale grating can be obtained as well as the out-of-flatness of the reference mirror in the Fizeau interferometer simultaneously. Figure 25a shows the setup for the measurement of the zeroth-order diffracted beam from the planar scale grating. The wavefront phase *I*_0_(*x*,*y*) can be expressed by using the out-of-flatness *e_Z_*(*x*,*y*) of the planar scale grating and that *e*_Ref_(*x*,*y*) of the reference mirror in the Fizeau interferometer as follows [86]:

(20) $$I_{0}(x,y)=\left(4\pi /\lambda \right)\left\{e_{Z}(x,y)-e_{\mathrm{ref}}(x,y)\right\}$$

where *λ* is the wavelength of the laser beam in the Fizeau interferometer.

The wavefront phases of the positive and negative first-order diffracted beams can be obtained by setting the planar scale grating in the Littrow configuration. Figure 25b shows a schematic of the setup in the Fizeau interferometer for the measurement of the wavefront phases of the positive and negative first-order diffracted beams. 

By rotating the planar scale grating about the *Y*-axis with an angle of ±*θ*/2, where *θ* is the positive first-order diffraction angle, the wavefront phases of the positive and negative first-order diffracted beams along the *X*-direction *I_X_*_+1_ and *I_X_*_-1_, respectively, can be obtained by using the Fizeau interferometer. *I_X_*_+1_ and *I_X_*_-1_ to be obtained by the Fizeau interferometer can be expressed as follows [86]:

(21) $$I_{X+1}=\left(2\pi /g\right)\cdot e_{X}(x,y)+\left(4\pi /\lambda \right)\cdot \left\{\;e_{Z}(x,y)\cdot \cos\left(\theta /2\right)-e_{\mathrm{ref}}(x,y)\right\}$$

(22) $$I_{X-1}=-\left(2\pi /g\right)\cdot e_{X}(x,y)+\left(4\pi /\lambda \right)\cdot \left\{\;e_{Z}(x,y)\cdot \cos\left(\theta /2\right)-e_{\mathrm{ref}}(x,y)\right\}$$

where *g* is the ideal grating pitch, *e_X_*(*x*,*y*) and *e_Y_*(*x*,*y*) are the pitch deviations at the position (*x*,*y*) along the *X*- and *Y*-directions, respectively. As can be seen in Figure 25a and the above equations, the grating pitch deviation *g_X_*(*x*,*y*) affects both *I_X_*_+1_ and *I_X_*_-1_, and can therefore be calculated through separating *e_Z_*(*x*,*y*) and *e*_ref_(*x*,*y*). In the same manner, the wavefront phases of the positive and negative first-order diffracted beam along the *Y*-axis *I_Y_*_+1_ and *I_Y_*_-1_, respectively, can also be obtained by the Fizeau interferometer, and can be expressed as follows [86]:

(23) $$I_{Y+1}=\left(2\pi /g\right)\cdot e_{Y}(x,y)+\left(4\pi /\lambda \right)\cdot \left\{\;e_{Z}(x,y)\cdot \cos\left(\theta /2\right)-e_{ref}(x,y)\right\}$$

(24) $$I_{Y-1}=-\left(2\pi /g\right)\cdot e_{Y}(x,y)+\left(4\pi /\lambda \right)\cdot \left\{\;e_{Z}(x,y)\cdot \cos\left(\theta /2\right)-e_{ref}(x,y)\right\}$$

From the above equations, *e_X_*(*x*,*y*), *e_Y_*(*x*,*y*), *e*_ref_(*x*,*y*) and *e_Z_*(*x*,*y*) can be obtained as follows [86]:

(25) $$e_{X}(x,y)=\left(g/4\pi \right)\left\{I_{X+1}(x,y)-I_{X-1}(x,y)\right\}$$

(26) $$e_{Y}(x,y)=\left(g/4\pi \right)\left\{I_{Y+1}(x,y)-I_{Y-1}(x,y)\right\}$$

(27) $$\begin{array}{ll} e_{\mathrm{Ref}}(x,y) & =\frac{\lambda }{8\pi \left(1-\cos(\theta /2)\right)}\left\{I_{X+1}(x,y)+I_{X-1}\;(x,y)+2I_{0}(x,y)\cos(\theta /2)\right\}\\ & =\frac{\lambda }{8\pi \left(1-\cos(\theta /2)\right)}\left\{I_{Y+1}\;(x,y)+I_{Y-1}\;(x,y)+2I_{0}(x,y)\cos(\theta /2)\right\} \end{array}$$

(28) $$\begin{array}{ll} e_{Z}(x,y) & =\frac{\lambda }{8\pi \left(1-\cos(\theta /2)\right)}\left\{I_{X+1}\;(x,y)+I_{X-1}\;(x,y)-2\cdot I_{0}(x,y)\right\}\\ & =\frac{\lambda }{8\pi \left(1-\cos(\theta /2)\right)}\left\{I_{Y+1}\;(x,y)+I_{Y-1}\;(x,y)-2\cdot I_{0}(x,y)\right\} \end{array}$$

As can be seen in the above equations, grating pitch deviations and the out-of-flatness of the planar scale grating, as well as the out-of-flatness of the reference mirror in the Fizeau interferometer, can be obtained simultaneously in short experiment time.

**Figure 26:**
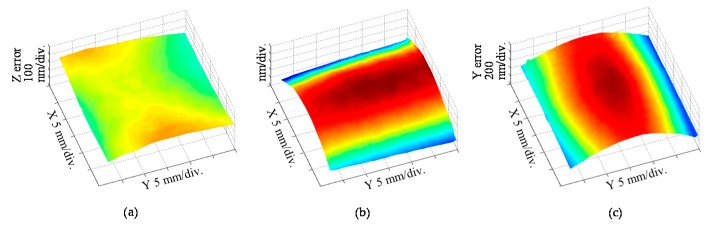
The evaluated large-area planar scale gratings [86]. (**a**) *Z*-directional out-of-flatness; (**b**) *X*-directional pitch deviation; (**c**) *Y*-directional pitch deviation.

Figure 26 shows the *X*- and *Y*-directional pitch deviations and the out-of-flatness of a planar scale grating evaluated by using a commercially-available Fizeau interferometer (ZYGO GPI-XP) [86]. The planar scale grating was placed in the field of view of the Fizeau interferometer while mounting it onto a two-axis manual tilt stage so that the planar scale grating could be set in the Littrow configurations. The required time for a whole experimental procedure was approximately five minutes. These experimental results have demonstrated the feasibility of evaluating the grating pitch deviations while separating from the out-of-flatness of the scale grating and the reference mirror in the Fizeau interferometer. The validity of the obtained grating pitch deviations has been confirmed through employing the planar scale grating in the three-axis planar encoder and comparing the measured displacement along the *X*- and *Y*-directions with those by the commercial laser interferometers [86]. 

**Figure 27:**
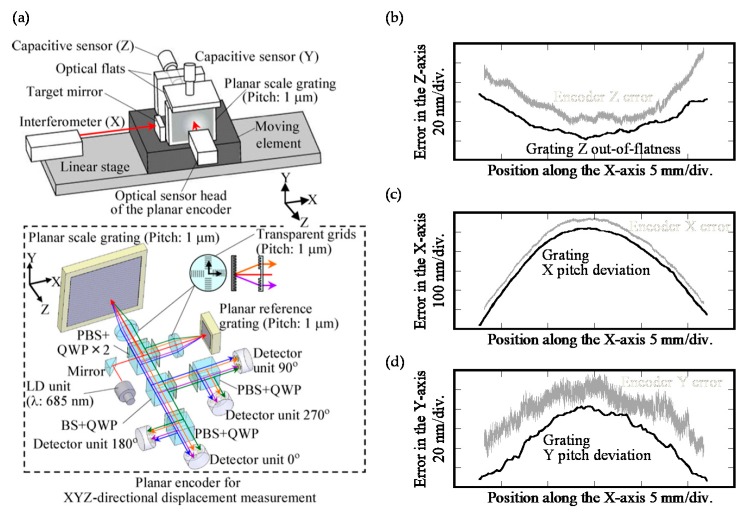
Verification of the obtained out-of-flatness and the pitch deviations [86]. (**a**) Experimental setup for evaluation of the measurement errors of the planar encoder; (**b**) *Z*-directional out-of-flatness; (**c**) *X*-directional pitch deviation; (**d**) *Y*-directional pitch deviation.

Figure 27a shows the experimental setup used for the experiment. Figure 27b shows the *Z*-directional position detection error in the planar encoder. In the figure, the cross-sectional profile of the corresponding *Z*-directional out-of-flatness of the planar scale grating shown in Figure 26a is also indicated. As can be seen in the figure, a good agreement can be found between the position detection error and the evaluated out-of-flatness of the planar scale grating. In the same manner, the *X*- and *Y*-directional measurement errors in the planar encoder were compared with the corresponding *X*-and *Y*-directional pitch deviation of the planar scale grating as shown in Figure 27c,d, respectively. From these results, the feasibility of the proposed fast evaluation method of the pitch deviation and out-of-flatness error of a planar scale grating has been verified.

## 5. Conclusions

In this review article, multi-axis optical sensors for precision dimensional metrology have been explained, while focusing on the three-axis planar encoder and the three-axis autocollimator that can measure three-axis translational displacement and the three-axis angular displacements, respectively, by employing a planar scale grating. In addition, a method of expanding the measurement range of the planar encoder by using a mosaic scale grating and a multi-beam optical sensor head is also explained. Furthermore, the six-degree-of-freedom planar encoder, which is capable of detecting three-dimensional translational displacement as well as the three-dimensional rotational displacement by using a single measurement laser beam, has been introduced. Since a planar scale grating is employed as the ruler for displacement measurement, the fabrication of planar scale grating, as well as the evaluation of the pitch deviation of the fabricated planar scale grating, is important tasks to be addressed. In this review article, the non-orthogonal two-axis Lloyd’s mirror interferometer, which is based on the principle of interference lithography, for the fabrication of large-scale planar scale gratings has been explained, as well as the pitch deviation measurement method based on the Fizeau interferometer.

A sub-nanometric resolution for measurement of linear displacement and a resolution better than 0.001 arc-second for measurement of angular displacement about a single axis have already been achieved by the conventional technologies. Meanwhile, when these technologies are applied for multi-axis displacement measurement, it suffers from the increase of measurement uncertainty due to the increase of the degrees of freedom to be measured. Since demands on further higher positioning accuracy in multi-axis positioning systems for precision dimensional metrology are increasing, it is necessary to improve the accuracy of multi-axis translational and angular displacement measurements. Furthermore, it is necessary to realize precision dimensional metrology traceable to the primary standard of length. Some attempts have already been made to realize multi-axis measurement directly traceable to the primary standard of length with the enhancement of a mode-locked femtosecond laser [146,147,148,149]. The multi-axis optical sensor technologies explained in this paper as well as the new measurement methods with the mode-locked femtosecond laser are expected to be a breakthrough technology in next-generation precision engineering.
